# Supplementary material for: Iron Deficiency Is Associated with Elevated Parathormone Levels, Low Vitamin D Status, and Risk of Bone Loss in Omnivores and Plant-Based Diet Consumers
Source: Int J Mol Sci. 2024 Sep 24;25(19):10290. doi: 10.3390/ijms251910290 (PMC11477403; doi:10.3390/ijms251910290)
Supplement: Supplementary file 1 [file ijms-25-10290-s001.zip › Figure S1.pdf]

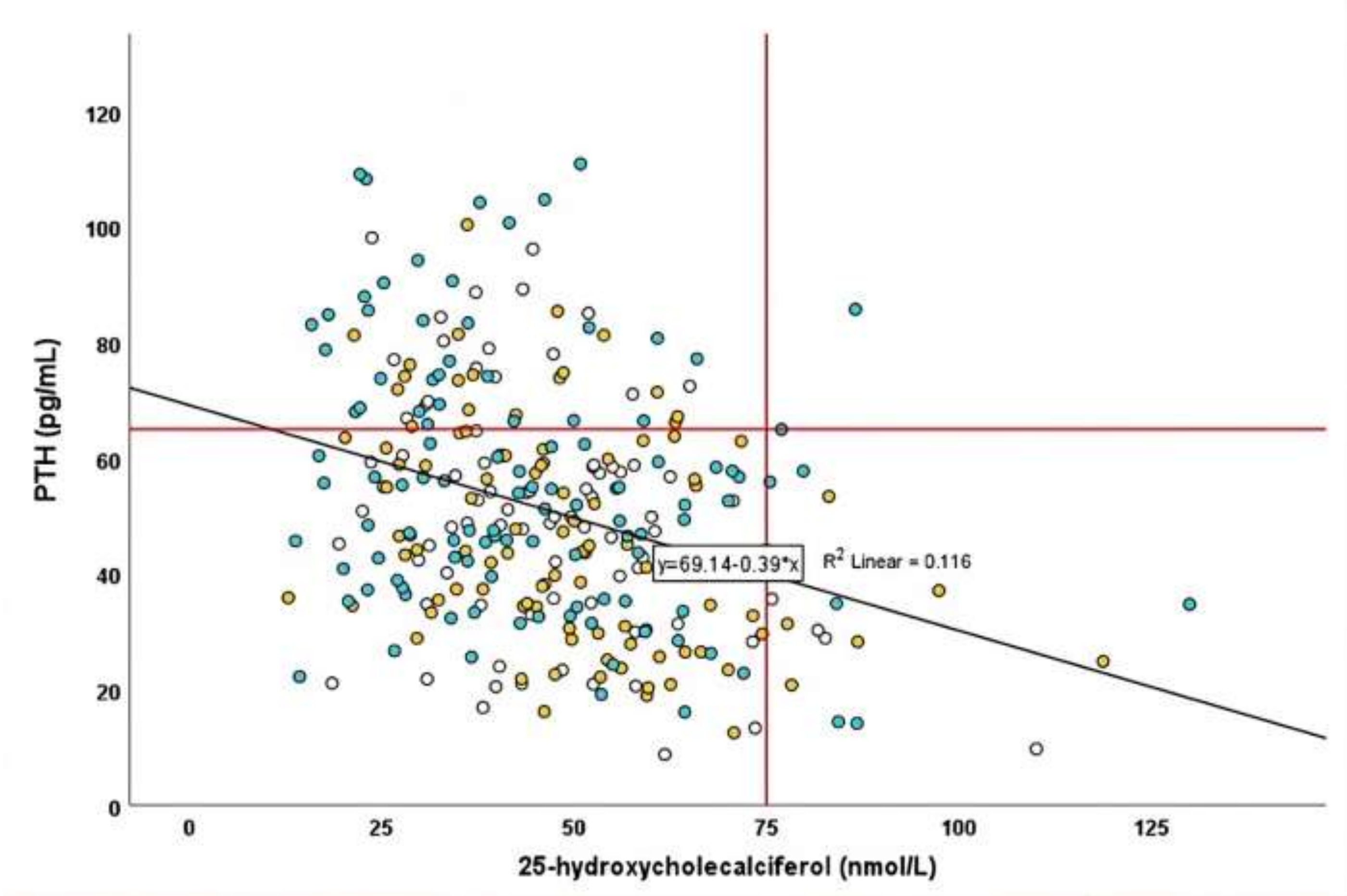

Figure S1. Linear regression between 25-hydroxycholecalciferol (25-OHD) and PTH levels. White circles, omnivores; yellow circles, lacto-ovo vegetarians; blue circles, vegans. Red lines indicate the cut-off values for vitamin D sufficiency (25-hydroxycholecalciferol, 75 nmol/L), and hyperparathyroidism (PTH, 65 pg/mL). The regression was significant with $p<0.001$.
The linear fit lines for the three diet groups were very close to each other and the fitted equations were similar (R^2 linear= 0.126, 0.141, and 0.087, for the omnivores, lacto-ovo vegetarians, and vegans, respectively).
