# Supplementary material for: Iron Deficiency Is Associated with Elevated Parathormone Levels, Low Vitamin D Status, and Risk of Bone Loss in Omnivores and Plant-Based Diet Consumers
Source: Int J Mol Sci. 2024 Sep 24;25(19):10290. doi: 10.3390/ijms251910290 (PMC11477403; doi:10.3390/ijms251910290)
Supplement: Supplementary file 1 [file ijms-25-10290-s001.zip › Tables S1 and S2.pdf]

Table S1. Correlations between PTH and bone remodeling biomarkers with iron status parameters

	Pearson Correlation	Sig. (2-tailed)	95% Confidence Intervals [a]	
			Lower	Upper
PTH - Hemoglobin	-0.073	0.208	-0.186	0.041
PTH - Serum iron	**0.196**	**0.001**	0.082	0.304
PTH - Transferrin	-0.032	0.586	-0.146	0.083
PTH - Transferrin saturation	-0.040	0.492	-0.154	0.075
PTH - Ferritin	-0.043	0.466	-0.156	0.072
PTH - 25-OHD	**-0.341**	**<0.001**	-0.438	-0.235
PTH - BAP	**0.176**	**0.003**	0.062	0.284
PTH - NTx	**0.211**	**<0.001**	0.098	0.319
Transferrin saturation - 25-OHD	-0.003	0.955	-0.117	0.111
Transferrin saturation - BAP	0.057	0.329	-0.058	0.170
Transferrin saturation - NTx	0.147	0.012	0.032	0.257
Ferritin - 25-OHD	-0.068	0.238	-0.180	0.045
Ferritin - BAP	0.056	0.333	-0.058	0.168
Ferritin - NTx	-0.044	0.455	-0.157	0.071
25-OHD - BAP	-0.107	0.066	-0.218	0.007
25-OHD - NTx	-0.060	0.306	-0.173	0.055
BAP - NTx	**0.188**	**0.001**	0.075	0.296

[a] Estimation is based on Fisher's r-to-z transformation with bias adjustment; *p* in bold indicate significant effects; PTH, parathormone; 25-OHD, 25-hydroxycholecalciferol; BAP, bone alkaline phosphatase; NTx, N-terminal telopeptide of collagen I.

Table S2. Hematological parameters of participants according to sex and PTH status

	**Normal-PTH (≤ 65 pg/mL)**			**High-PTH (>65 pg/mL)**				
	Man n=75	Woman n=153	All n=228	Man n=23	Woman n=46	All n=69	*p* sex	*p* PTH status
MCV (fL)	89.8 ± 5.0	90.4 ± 5.5	90.2 ± 5.4	88.3 ± 4.6	90.1 ± 6.4	89.5 ± 5.9	0.121	0.247
RDW (%)	13.1 ± 0.8	13.5 ± 1.3	13.4 ± 1.2	13.4 ± 1.2	13.8 ± 1.5	13.7 ± 1.4	**0.042**	0.113
MCH (pg)	30.6 ± 1.9	30.1 ± 2.2	30.3 ± 2.1	29.9 ± 1.8	30.3 ± 2.7	30.2 ± 2.4	0.969	0.410
MCHC (g/dL)	34.1 ± 0.9	33.3 ± 1.1	33.6 ± 1.1	33.9 ± 0.8	33.6 ± 0.9	33.7 ± 0.9	**<0.001**	0.817
Platelets (10^3/μL)	215 ± 49	245 ± 64	236 ± 61	196 ± 40	243 ± 50	228 ± 51	**<0.001**	0.186
MPV (fL)	9.3 ± 0.8	9.7 ± 1.2	9.5 ± 1.1	9.5 ± 0.8	9.3 ± 1.0	9.4 ± 1.0	0.584	0.648

Values are mean ± SD; *p* in bold indicate significant effects. PTH, parathormone; MCV, mean corpuscular volume; RDW, red cell distribution width; MCH, mean corpuscular hemoglobin; MCHC, mean corpuscular hemoglobin concentration; MPV, mean platelet volume. There were no significant sex-PTH status interactions.
